# Germination Improves the Antioxidant and Anti-Melanogenic Properties of Resveratrol-Enriched DJ526 Rice in Melan-a Cells

**DOI:** 10.3390/cimb48101004

**Published:** 2026-09-29

**Authors:** Aimee Carol Tangonan, Sun Yeou Kim, So-Hyeon Baek

**Affiliations:** 1 Department of Agricultural Life Science, Sunchon National University, Suncheon 57922, Republic of Korea; aicatan3792@gmail.com; 2 Laboratory of Pharmacognosy, College of Pharmacy, Gachon University, Incheon 21936, Republic of Korea; sunnykim@gachon.ac.kr

**Keywords:** resveratrol-enriched rice, seed germination, hyperpigmentation, tyrosinase inhibitor, MITF, cosmeceuticals

## Abstract

Resveratrol is a natural polyphenolic compound with antioxidant and anti-melanogenic activities, but it occurs only at low levels in most edible plant tissues, motivating the development of enriched sources. Resveratrol-enriched DJ526 rice provides such a source, and seed germination may further modulate its biological activity. Therefore, this study investigated the effects of 5-day germination on the antioxidant and anti-melanogenic properties of resveratrol-enriched DJ526 rice in Melan-a cells. Seed extracts of ungerminated DJ526 (DJ526_0), germinated DJ526 (DJ526_5), and non-transgenic controls (DJ_0 and DJ_5) were evaluated for antioxidant capacity, cell viability, melanogenic activities, and the expression of MITF and its downstream targets. Five-day germination increased piceid and resveratrol content in DJ526_5 from 4.73 ± 0.06 to 16.90 ± 0.00 µg/g dry weight and from 2.60 ± 0.00 to 3.27 ± 0.06 µg/g dry weight, respectively. DJ526_5 exhibited the strongest antioxidant capacity (ABTS IC_50_, 16.81 ± 1.26 mg/mL). At 100 µg/mL, DJ526_5 reduced intracellular melanin content and tyrosinase activity to 59.48 ± 2.13% and 45.63 ± 0.24% of the control, respectively, without reducing cell viability. DJ526_5 also reduced the mRNA and protein expression of MITF, tyrosinase, TRP-1, and TRP-2. These findings support the potential of DJ526 as a functional ingredient for hyperpigmentation management.

## 1. Introduction

Control of skin pigmentation has attracted considerable global interest, as pigmentary disorders impose substantial aesthetic and psychosocial burdens on those affected [1,2]. This concern is mirrored in the marketplace, where demand for skin-whitening cosmetics continues to rise, driven largely by cultural associations between lighter skin tone and beauty, particularly across Asian markets [3,4]. Skin coloration is regulated by intrinsic factors, including genetic background and skin phototype, together with extrinsic stimuli such as UV radiation, hormonal fluctuations, and environmental pollution [5,6,7]. Although melanin provides essential photoprotection by absorbing and scattering UV radiation, its overproduction precipitates clinically significant hyperpigmentation disorders, including melasma, solar lentigines, ephelides, and post-inflammatory hyperpigmentation [1,8].

At the molecular core of pigment biosynthesis lies tyrosinase, the rate-limiting, copper-containing metalloenzyme that catalyzes the hydroxylation of L-tyrosine to L-DOPA and its subsequent oxidation to dopaquinone [9]. Downstream, tyrosinase-related proteins 1 and 2 (TRP-1, TRP-2) direct the production of eumelanin and pheomelanin under the transcriptional control of the microphthalmia-associated transcription factor (MITF) [10]. Conventional depigmenting agents such as hydroquinone, corticosteroids, and kojic acid target these pathways but are associated with serious adverse effects, including contact dermatitis, ochronosis, skin atrophy, and systemic toxicity with prolonged use [11,12]. These limitations have intensified the search for naturally derived compounds that selectively suppress tyrosinase activity without inducing cytotoxicity in normal melanocytes [13,14].

Plant-derived phytochemicals with reported depigmenting or anti-melanogenic activity include flavonoids, phenolic acids, terpenoids, and other polyphenolic compounds [15]. Well-recognized examples include arbutin, a hydroquinone glucoside from bearberry and other plants that inhibits melanosomal tyrosinase [16,17,18]; aloesin from *Aloe vera*, which inhibits tyrosinase activity and has been reported to reduce UV-induced hyperpigmentation [18,19]; glabridin, a licorice-derived flavonoid that inhibits tyrosinase activity and melanogenesis [20]; and ellagic acid, a polyphenol found in fruits and nuts that suppresses melanogenesis through tyrosinase inhibition and copper chelation [21]. Additional reported anti-melanogenic phytochemicals include galangin, ferulic acid, *p*-coumaric acid, and ginsenosides [22]. These phytochemicals may suppress melanogenesis through direct inhibition of tyrosinase activity, attenuation of oxidative stress, and/or regulation of MITF and its downstream targets, including tyrosinase, TRP-1, and TRP-2 [18,19,23].

Resveratrol (3,5,4′-trihydroxy-trans-stilbene) is a naturally occurring stilbene polyphenol found in several edible plant sources, particularly grapes and grape-derived products [24,25]. It exhibits antioxidant properties through radical-scavenging activity and modulation of cellular redox-regulatory pathways. Because oxidative stress is implicated in melanogenesis-related signaling, these antioxidant properties may also be relevant to pigmentation control [26,27,28,29]. In addition, resveratrol has been reported to suppress melanin production through direct inhibition of tyrosinase activity and downregulation of MITF and its downstream targets, including tyrosinase, TRP-1, and TRP-2 [30,31,32,33]. As MITF coordinates the transcriptional program responsible for melanocyte differentiation and melanin synthesis, its suppression provides a mechanistic basis for the anti-melanogenic activity of resveratrol. Collectively, these properties highlight resveratrol as a potential natural candidate for the management of excessive pigmentation.

A promising strategy for maximizing the bioactive content of such compounds is to harness early plant developmental stages, since sprouts and microgreens exhibit markedly higher phytochemical densities and antioxidant capacities than mature tissues. Germination drives this enhancement by reactivating seed metabolism, stimulating secondary metabolite biosynthesis, and degrading anti-nutritional factors that restrict bioavailability [34,35]. Consistent with this, short-term germination has been shown to substantially increase phenolics, flavonoids, and antioxidant capacity across multiple species, including buckwheat [36], wheat [37], chickpea [38], and peanut [39]. Notably, germinated riceberry rice yielded extracts with elevated phenolic acids and enhanced tyrosinase inhibition and melanogenesis suppression in B16 melanoma cells [40]. Germination can improve the functional properties of rice, although the magnitude and profile of phytochemical changes vary among genotypes [41,42]. In addition to germinated riceberry rice, fermented rice bran, fermented unpolished black rice, proanthocyanidin-rich red rice, and polyphenol- and flavonoid-enriched rice seed extracts have shown anti-melanogenic activity through inhibition of tyrosinase activity and/or MITF-associated signaling [43,44,45,46]. In contrast, the groundnut (*Arachis hypogaea*) *resveratrol synthase 3* (*AhRS3*) gene was introduced into the Dongjin background to generate the independent resveratrol-producing lines Iksan515 and Iksan526; Iksan526 accumulated more resveratrol and piceid and was selected as the DJ526 platform [47,48]. Germination of DJ526 may therefore provide a distinct strategy to increase its stilbene pool and associated antioxidant and anti-melanogenic potential.

The encoded resveratrol synthase *AhRS3* can direct endogenous rice phenylpropanoid precursors, *p*-coumaroyl-CoA and malonyl-CoA, toward trans-resveratrol production [48,49]. In plant tissues, resveratrol may subsequently be glycosylated by endogenous UDP-glycosyltransferases (UGTs) to form piceid, a glucosylated derivative that contributes to the total stilbene pool (Figure S1) [50]. Previous studies have demonstrated anti-melanogenic activity of resveratrol-enriched rice-derived materials in cellular and in vivo pigmentation models [46,51]. Moreover, germination of DJ526 has been reported to increase both resveratrol and piceid contents and to enhance antioxidant, anti-inflammatory and anti-adipogenic activities in macrophage and adipocyte models relative to ungerminated material [46,51]. However, these previous studies did not determine whether short-term germination enhances the anti-melanogenic activity of DJ526 seed extract in melanocytes. We therefore hypothesized that germination-associated enrichment of resveratrol and piceid may contribute to enhanced antioxidant and anti-melanogenic activities of DJ526. Therefore, the present study evaluates the anti-melanogenic efficacy and cellular bioactivity of germinated DJ526 extracts, establishing their potential as functional ingredients for the management of hyperpigmentation disorders.

## 2. Materials and Methods

### 2.1. Sample Preparation and Extraction

Test materials were prepared following a previously reported procedure [27]. Seeds of DJ526 and the control line DJ were dehulled, surface-sterilized by sequential washing with 70% ethanol (Daejung Chemicals & Metals Co., Ltd., Siheung, Republic of Korea) and 3% sodium hypochlorite (Daejung Chemicals & Metals Co., Ltd., Siheung, Republic of Korea), and rinsed several times with distilled water to remove residual disinfectants. Sterilized seeds were germinated in sterile distilled water for 5 days. This germination duration was selected based on previous studies of Iksan 526/DJ526 reporting increased trans-resveratrol and piceid contents after 5 days of germination, as well as a distinct shift in the resveratrol/piceid balance during germination [50,52]. Both germinated and ungerminated seeds were pulverized prior to extraction.

For each sample, 5 g of seed powder was extracted in 50 mL of 80% methanol (Merck KGaA, Darmstadt, Germany). The suspensions were sonicated at 40 °C for 60 min, filtered through filter paper (CHMLAB Group, Barcelona, Spain), and concentrated using a rotary evaporator with a water-bath temperature of 50 °C. Residual solvent was removed by drying the concentrate overnight in a centrifugal vacuum concentrator (HyperVAC, Gyrozen Co., Ltd., Daejeon, Republic of Korea) to obtain dry crude extracts. Each extract was weighed and dissolved in dimethyl sulfoxide (DMSO; Sigma-Aldrich, St. Louis, MO, USA) to prepare stock solutions of 10, 25, 50, and 100 mg/mL. For cell-based assays, the extracts were diluted in complete culture medium to final concentrations of 10, 25, 50, and 100 µg/mL. Accordingly, the final DMSO concentration was 0.1% (*v*/*v*) in all extract-treated groups. Complete culture medium containing 0.1% (*v*/*v*) DMSO without extract was used as the vehicle control in all cell-based experiments. Arbutin (Sigma-Aldrich, St. Louis, MO, USA) was used as the positive control [29,30].

### 2.2. Determination of Piceid and Resveratrol Contents

The piceid and resveratrol contents of germinated and ungerminated rice seed extracts were determined by high-performance liquid chromatography (HPLC). Analyses were performed using a Waters e2695 separation module (Waters Pacific Pte. Ltd., Singapore) equipped with a C18 reverse-phase column (4.6 mm × 150 mm; Waters, Milford, MA, USA). Pulverized seed samples were extracted with 80% methanol by sonication for 60 min at 40 °C. The supernatants were collected and filtered through PTFE-H 0.2 μm membrane filter (Hyundai Micro, Seoul, Republic of Korea) prior to HPLC analysis.

Chromatographic separation was performed using distilled water (solvent A) and acetonitrile (solvent B) as the mobile phases. The gradient elution program was as follows: 10% B for 0–37 min, increased to 30% B from 37–38 min, increased to 100% B from 38–45 min, and returned to 10% B from 45–50 min. The flow rate was maintained at 1.0 mL/min, and the injection volume was 10 μL. Eluted compounds were monitored at 308 nm. Chromatographic data were acquired and processed using Empower 2 chromatography software (Waters Corporation, Milford, MA, USA). Piceid and resveratrol were identified and quantified by comparison with their respective authentic standard calibration curves. The HPLC chromatographic profiles and the quantified piceid and resveratrol contents of the extracts used in the present study are presented in Figure S2.

### 2.3. ABTS Reduction Assay

The antioxidant capacity of the extracts was evaluated using the spectrophotometric ABTS reduction assay [53]. Radical generation followed a previously reported protocol [54]. A 7 mM ABTS stock (Roche, Basel, Switzerland) was combined with 2.45 mM potassium persulfate (Sigma-Aldrich, St. Louis, MO, USA) at a 1:1 ratio and kept in the dark at room temperature for 12 to 16 h to form the ABTS^+^ radical cation. The resulting solution was diluted in absolute ethanol until it reached an absorbance of 0.700 ± 0.02 at 734 nm, and this preparation served as the working reagent for the decolorization assay.

To perform the assay, 10 µL of each sample, prepared at 10, 25, 50, and 100 mg/mL in DMSO, vitamin C standards or DMSO (control vehicle), was placed in a 96-well plate and combined with 190 µL of the ABTS^+^ working solution. The final reaction-mixture concentrations of extracts were 0.50, 1.25, 2.50, and 5.00 mg/mL, respectively. Plates were protected from light and incubated for 7 min before absorbance was recorded at 734 nm. For control wells, deionized water replaced the sample. ABTS radical scavenging ability (%) was obtained using the following formula:

(1) $$ABTS\;radical\;scavenging\;activity\;(\%)=\frac{{OD}_{734}\;of\;control-{OD}_{734}\;of\;treatment}{{OD}_{734}\;of\;control}\times 100$$

Ascorbic acid (Sigma-Aldrich, St. Louis, MO, USA) was prepared in deionized water across a range of 3.91 to 250.00 µg/mL and used as the reference standard for vitamin C equivalent antioxidant capacity (VCEAC) (Figure S3). VCEAC was determined using the following formula:

(2) $$VCEAC\;(\mathrm{mg}\;\mathrm{VCE}/\mathrm{mL})=\;\frac{y-b}{1000\times m},$$

where:

*y*: ABTS scavenging activity (%) of the treatment;

*b*: y-intercept of the vitamin C standard curve;

*m*: slope of the vitamin C standard curve;

1/1000: converts µg VCE/mL to mg VCE/mL.

The IC_50_, defined as the concentration required to lower radical activity by 50%, was likewise derived from the dose–response curve obtained by plotting the ABTS scavenging activity of each treatment.

### 2.4. Cell Culture Preparation

Melan-a cells, an immortalized murine melanocyte cell line, were kindly provided by Prof. Sun Yeou Kim (College of Pharmacy, Gachon University, Incheon, Republic of Korea). Melan-a cells were grown in expansion medium based on RPMI 1640 (Welgene, Gyeongsan, Republic of Korea) supplemented with 10% Fetal Bovine Serum (Gibco, Thermo Fisher Scientific, Inc., Waltham, MA, USA), 1% penicillin–streptomycin (Hyclone Laboratories, Inc., Logan, UT, USA), and 200 nM 12-O-tetradecanoylphorbol-13-acetate (TPA; Sigma-Aldrich, St. Louis, MO, USA). Cultures were kept at 37 °C in a humidified 5% CO_2_ atmosphere and propagated to roughly 70% confluency prior to use.

### 2.5. Cell Viability Assay

Aliquots of Melan-a cells (1 × 10^4^ per well) were distributed across a 96-well plate in expansion medium and left to settle for 24 h in a 37 °C, 5% CO_2_ incubator. The spent medium was replaced with expansion medium carrying the treatments, and the plate was returned to the incubator for another 72 h under identical settings. At the end of the treatment window, the medium was aspirated, and each well received 110 µL of EZ-Cytox reagent (DoGenBio, Seoul, Republic of Korea) prepared as a 10% (*v*/*v*) dilution in 1× DPBS (Welgene, Gyeongsan, Republic of Korea), after which the plate was incubated for 4 h at 37 °C.

Subsequently, a 100 µL aliquot of each reaction was moved to a clean plate, and its absorbance was recorded at 450 nm with a SpectraMax^®^ ABS Plus microplate reader (Molecular Devices, LLC, San Jose, CA, USA). Cell viability was calculated using the following formula:

(3) $$Cell\;viability\;(\%)=\frac{{OD}_{450}\;of\;treatment-{OD}_{450}\;of\;blank}{{OD}_{450}\;of\;control-{OD}_{450}\;of\;blank}\times 100.$$

### 2.6. Intracellular Tyrosinase Activity

Cellular tyrosinase activity was evaluated using L-DOPA (Sigma-Aldrich, St. Louis, MO, USA) as the substrate [55]. A 24-well plate was loaded with Melan-a cells at 5 × 10^4^ cells per well in 500 µL of expansion medium and incubated for 24 h. Fresh medium containing the treatments then replaced the original medium, and the cells were cultured for an additional 72 h.

After incubation, the cells were washed with 1× PBS, collected, and lysed in 0.1 M sodium phosphate buffer containing 0.1% Triton X-100 (R&D Systems, Inc., Minneapolis, MN, USA). Protein concentration was determined by the BCA assay (iNtRON Biotechnology, Inc., Seongnam, Republic of Korea) with bovine serum albumin (BSA) as the standard for quantification (Figure S4). An aliquot containing 40 µg of protein in an 80 µL volume was combined with 20 µL of 2 mg/mL L-DOPA and incubated at 37 °C for 3 h. Absorbance was then read at 475 nm on a spectrophotometer, and cellular tyrosinase activity was determined using the following formula:

(4) $$Relative\;tyrosinase\;activity\;(\%)=\frac{{OD}_{475}\;of\;treatment}{{OD}_{475}\;of\;control}\times 100.$$

### 2.7. L-DOPA Staining

Melan-a cells were plated in 96-well plates at 1 × 10^4^ cells per well and given 24 h to adhere. The treatments were subsequently introduced, and the cultures were maintained for 72 h. Following this period, the cells were fixed in 4% paraformaldehyde (Daejung Chemicals & Metals Co., Ltd., Siheung, Republic of Korea) for 20 min, rinsed, and exposed to 2 mg/mL L-DOPA for 3 h at 37 °C. After two further PBS washes, the resulting staining was captured and analyzed on an IM-3 series microscope (Optika, Bergamo, Italy).

### 2.8. Intracellular Melanin Content Determination

Intracellular melanin was quantified following an earlier report [46]. For this purpose, 6-well plates were seeded with Melan-a cells at 5 × 10^5^ cells per well in 2000 µL of expansion medium, and the cells were given 24 h at 37 °C to adhere. Treatment medium then took the place of the growth medium, and the cultures were maintained for a further 72 h. Following treatment, the cells were detached, rinsed, and adjusted to equal numbers. Each pellet was diluted in 1 N NaOH (Merck KGaA, Darmstadt, Germany) supplemented with 10% DMSO and warmed in a heating block at 80 °C for 1 h. The absorbance of the dissolved melanin was determined at 405 nm on a SpectraMax^®^ ABS Plus microplate reader (Molecular Devices, LLC, San Jose, CA, USA), and melanin content was derived using the following formula:

(5) $$Relative\;melanin\;content\;(\%)=\frac{{OD}_{405}\;of\;treatment}{{OD}_{405}\;of\;control}\times 100.$$

### 2.9. Fontana–Masson Staining

Melan-a cells were dispensed into a 96-well plate at a density of 1 × 10^4^ cells per well and held for 24 h at 37 °C in 5% CO_2_ to permit attachment. The adherent cells were exposed to the treatments and cultured for a further 72 h. Afterward, cells were rinsed twice in 1× PBS, fixed with 10% buffered formalin for 30 min, and rinsed once more. Fontana–Masson staining (BIOGNOST, Ltd., Zagreb, Croatia) was performed in line with the manufacturer’s protocol.

Briefly, the cells were dehydrated in 60% isopropanol and treated with silver ammonia reagent supplied with the staining kit at 56 °C for 30 min. The wells were rinsed, after which 100 µL of 5% sodium thiosulfate was added. Following another wash, nuclear fast red was applied as a counterstain. The silver ammonia reagent, sodium thiosulfate, and nuclear fast red were all components of the staining kit. Excess stain was removed, the preparations were dried, and the stained cells were examined under a microscope (Optika, Bergamo, Italy). In situ melanin in Melan-a cells was quantified in ImageJ (version 1.54g; National Institutes of Health (NIH), Bethesda, MD, USA) [56] following a previously reported workflow [57,58] with minor modifications [59]. Total pigmented area was then obtained with the Analyze Particles function and expressed as the area fraction (% Area) of the total field. The relative melanin-stained area of each treatment was then calculated against the control group using the formula:

(6) $$Relative\;melanin\;stained\;area\;(\%)=\frac{\%\;Area\;of\;treatment}{\%\;Area\;of\;control}\times 100.$$

The Fontana–Masson-stained cells were further assessed morphologically. For each treatment group, three microscopic fields were analyzed in each of three independent biological experiments. In each field, 100 melanin-containing cells were evaluated and classified into four morphological grades (1+, 2+, 3+, and 4+) according to previously described criteria (Table 1) [40].

The classification was based on cell shape and branching pattern, cell-size category, and visible intracellular Fontana–Masson-positive pigment distribution. Images were anonymized before assessment, and the evaluator was blinded to treatment allocation to minimize subjective bias. The 1+–4+ classification was used as a semi-quantitative descriptive morphology/pigmentation assessment and was interpreted together with the ImageJ -based (version 1.54g; NIH) quantification of the Fontana–Masson-positive stained area.

### 2.10. RNA Extraction and Gene Expression Analysis

TRI Reagent (Invitrogen, Waltham, MA, USA) was used to recover total RNA in accordance with a previously described procedure [60]. The purity and yield of the extracted RNA were verified on a SpectraMax ABS Plus microplate reader (Molecular Devices, San Jose, CA, USA) by measuring the absorbance at 260/280 nm and 260/230 nm. RNA samples with A260/A280 and A260/A230 ratios between 1.8 and 2.0 were used for subsequent cDNA synthesis. For cDNA synthesis, 1 µg of RNA served as template in the AccuPower RocketScript RT Master Mix, RNase H Minus cDNA synthesis kit (Bioneer Corp, Seongnam, Republic of Korea). Guided by the measured RNA concentration, the resulting cDNA was brought to 5 ng/µL with nuclease-free water.

Melanogenesis-related transcripts were measured on a CFX Connect Real-Time PCR system (Bio-Rad, Hercules, CA, USA). Every 20 µL reaction was assembled with AccuPower GreenStar qPCR PreMix (Bioneer Corp, Seongnam, Republic of Korea), 10 µM of each gene-specific primer (Table S1), and 5 ng of cDNA template. Cycling proceeded under conditions reported earlier [46]. The amplification program consisted of an initial denaturation step at 95 °C for 10 min, followed by 40 cycles of denaturation at 95 °C for 20 s; annealing at 60 °C for 20 s; extension at 72 °C for 30 s; and final extension at 72 °C for 5 min. Product specificity was assessed by melt-curve analysis over the range of 65–95 °C. Transcript levels were normalized to GAPDH, and relative expression was calculated using the 2^−ΔΔCt^ method, with the Media group set as the calibrator.

### 2.11. Western Blot Analysis

For protein recovery, treated cells were disrupted in RIPA buffer containing 1× protease inhibitor cocktail (Quartett, Berlin, Germany) and kept on ice for 30 min. Spinning the lysates at 13,000 rpm for 30 min at 4 °C cleared the debris, and the supernatant was collected into a fresh microcentrifuge tube. Total protein was quantified by the BCA method against a BSA standard.

From each sample, 30 µg of protein was loaded and separated by SDS-PAGE before transfer to a PVDF membrane. The membranes were blocked with 5% (*w*/*v*) skim milk (BD Difco, Sparks, MD, USA) in 1x Tris-buffered saline containing 0.1% Tween-20 (TBST) for 2 h at room temperature and then probed overnight at 4 °C with primary antibodies directed against MITF (1:1000; Cell Signaling Technology, Danvers, MA, USA; Cat No. 97800), tyrosinase (1:100; Santa Cruz Biotechnology, Dallas, TX, USA; Cat No. sc-20035), TRP-1 (1:1000; Santa Cruz Biotechnology, Dallas, TX, USA; Cat No. sc-166857), TRP-2 (1:100; Santa Cruz Biotechnology, Dallas, TX, USA; Cat No. sc-74439), and GAPDH (1:1000; Santa Cruz Biotechnology, Dallas, TX, USA; Cat No. sc-32233). The blots were washed and then exposed to specific secondary antibody (goat anti-rabbit IgG; 1:2000; Cell Signaling Technology, Danvers, MA, USA; Cat No. 7074 or m-IgGκ BP-HRP; 1:2000; Santa Cruz Biotechnology, Dallas, TX, USA; Cat No. sc-516102) for 2 h at room temperature. Bands were developed with Pierce ECL Plus Western blotting substrate (Thermo Scientific, Waltham, MA, USA), imaged on a ChemiDoc Imaging System, and analyzed using Image Lab software (version 6.0.0; Bio-Rad, Hercules, CA, USA). The intensity of each target-protein band was normalized to the intensity of the corresponding GAPDH band, and the normalized values were expressed relative to the media-only control group.

### 2.12. Statistical Analysis

Results are reported as the mean ± standard deviation. All statistical computations were performed using IBM SPSS Statistics version 23. For multi-group comparisons, one-way analysis of variance (ANOVA) followed by Tukey’s post hoc test was applied. A threshold of *p* < 0.05 denoted statistical significance.

## 3. Results

### 3.1. HPLC Determination of Piceid and Resveratrol Contents

HPLC analysis was conducted to characterize the piceid and resveratrol contents of ungerminated and germinated rice extracts. Representative chromatograms and the complete quantitative comparison are presented in Figure S2. Piceid and resveratrol were not detected in the non-transgenic DJ_0 and DJ_5 extracts. In contrast, DJ526_0 contained 4.73 ± 0.06 µg/g dry weight piceid and 2.60 ± 0.00 µg/g dry weight resveratrol. After 5 days of germination, DJ526_5 contained 16.90 ± 0.00 µg/g dry weight piceid and 3.27 ± 0.06 µg/g dry weight resveratrol. Compared with DJ526_0, DJ526_5 exhibited significantly higher piceid and resveratrol contents (*p* < 0.001), corresponding to approximately 3.57-fold and 1.26-fold increases, respectively. Therefore, 5-day germination significantly enhanced the accumulation of both stilbene compounds in DJ526.

### 3.2. Antioxidant Capacity Determined by ABTS Assay

The antioxidant potential of germinated and ungerminated rice extracts was quantitatively assessed via ABTS radical scavenging activity and expressed as Vitamin C Equivalent Antioxidant Capacity (VCEAC). As illustrated in Table 2, all rice extracts demonstrated a dose-dependent increase in scavenging activity within the concentration range of 10 to 100 mg/mL. Among the tested groups, the germinated transgenic variety (DJ526_5) emerged as the most potent treatment, with scavenging capacity ranging from 35.28 ± 1.21% to 93.17 ± 0.18% and yielding the lowest IC_50_ value of 16.81 mg/mL. Notably, at the same concentration of 100 mg/mL, the scavenging percentage and VCEAC (0.243 mg VCE/mL) of DJ526_5 were comparable to that of arbutin (*p* > 0.05).

A consistent trend was observed where germination enhanced the antioxidant profile of both transgenic (DJ526) and parental (DJ) varieties. While the parental lines (DJ_0 and DJ_5) showed significant incremental improvements upon germination, their IC_50_ values remained beyond the tested range (>100 mg/mL). This highlights the superior baseline antioxidant capacity of the resveratrol-enriched DJ526 line. To validate the experimental system, DMSO was used as the vehicle control and exhibited low activity (3.97 ± 0.2%), confirming that the observed radical scavenging effects were derived specifically from the bioactive components within the rice extracts.

### 3.3. Cell Viability of Melan-a Cells Following Treatment with Germinated and Ungerminated Seed Extracts (EZ-Cytox Assay)

**Figure 1:**
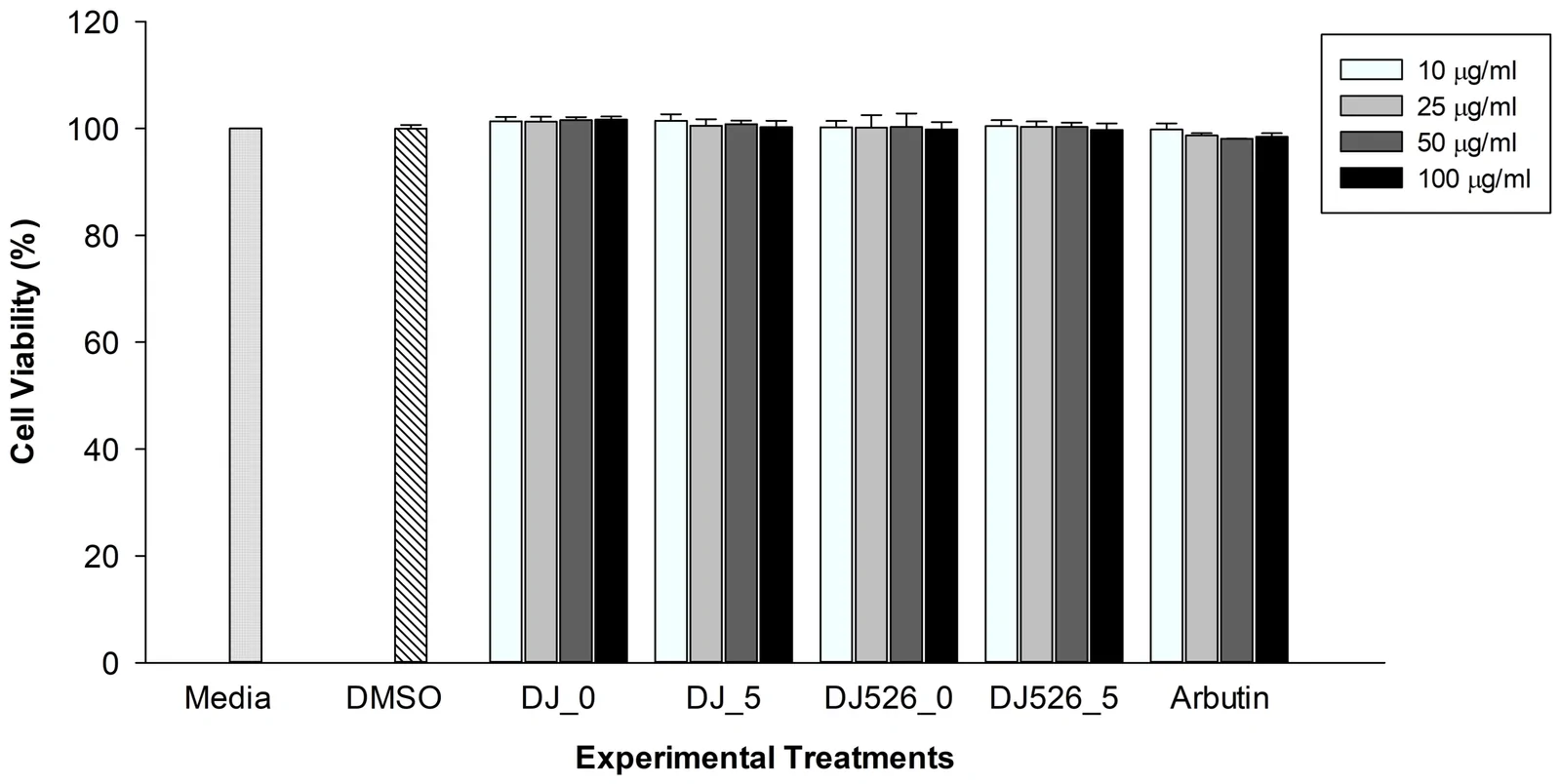
Effects of different concentrations of rice seed extracts on Melan-a cell viability. Data are presented as mean ± SD.

To ensure that the observed biological effects were not due to nonspecific cytotoxicity, Melan-a cell viability was assessed across a concentration range of 10–100 µg/mL (Figure 1). Cell viability remained consistently close to 100% with no significant differences compared to the medium control (*p* > 0.05), confirming that these concentrations were non-cytotoxic and suitable for subsequent melanogenic assays. Because 100 µg/mL was the highest tested concentration that did not significantly affect Melan-a cell viability, it was selected for subsequent anti-melanogenic assays; this selection was consistent with the concentration-dependent antioxidant activity observed for the extracts in the ABTS assay.

### 3.4. Intracellular Melanin Content and Tyrosinase Activity Following Treatment with DJ526_5

**Figure 2:**
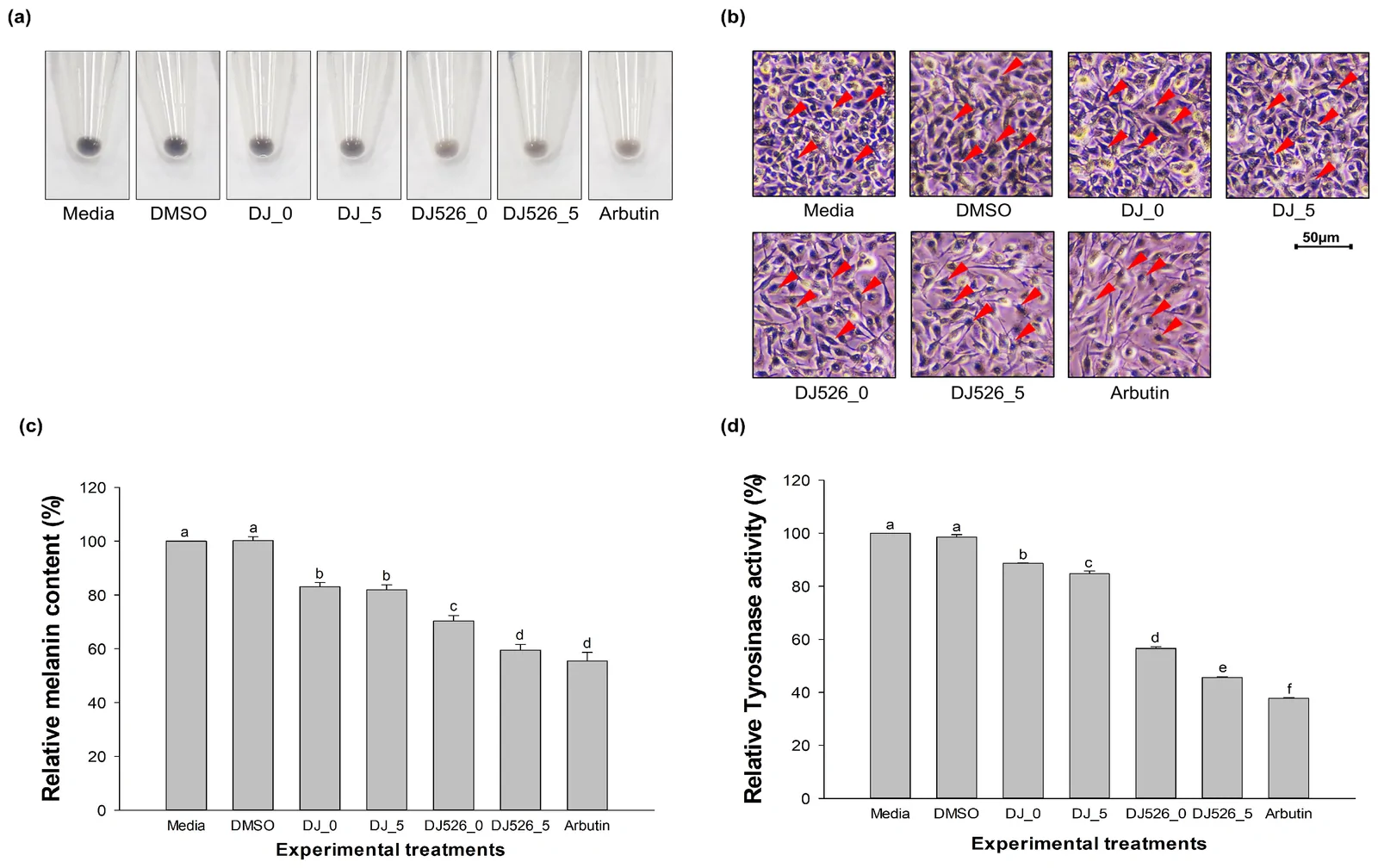
Effect of DJ526_5 treatments on melanin content and tyrosinase activity in Melan-a cells. (**a**) Representative images of Melan-a cell pellets after treatment; cell pellets were dissolved in 1 N NaOH for melanin quantification. (**b**) Representative images of L-DOPA-stained Melan-a cells after treatment. Red arrows indicate L-DOPA-stained melanin-containing spots. (**c**) Relative melanin content (%) of Melan-a cells after treatment. (**d**) Relative tyrosinase activity (%) of Melan-a cells after treatment. The concentrations of DMSO and the treatments were 0.1% (*v*/*v*) and 100 µg/mL, respectively. Values are presented as mean ± SD. Different lowercase letters (a–f) indicate statistically significant differences among treatments (*p* < 0.05); “a” generally corresponds to the highest content or activity, whereas letters b–f indicate progressively lower observations.

The anti-melanogenic activity of DJ526-based rice extracts was evidenced by reduced pellet pigmentation, melanin content, and cellular tyrosinase activity in Melan-a cells (Figure 2). Representative pellet images (Figure 2a) showed visibly lighter pigmentation in DJ526-based treatments than in the DMSO and RPMI controls, indicating suppressed melanin accumulation. Likewise, the in situ L-DOPA staining images (Figure 2b) showed fewer and lighter melanin-containing spots in treated cells, supporting reduced tyrosinase activity at the cellular level.

Relative to RPMI, DJ526_5 reduced melanin content to 59.48 ± 2.13% and tyrosinase activity to 45.63 ± 0.24%, whereas DJ526_0 showed values of 70.32 ± 2.04% and 56.59 ± 0.65%, respectively (Figure 2c,d). Arbutin remained the most active treatment, although DJ526_5 showed comparable inhibition. The enhanced inhibitory effects of DJ526_5 were observed together with the germination-associated increases in piceid and resveratrol contents described in Section 3.1.

### 3.5. Fontana–Masson Staining: Relative Stained Area and Morphological Assessment

**Figure 3:**
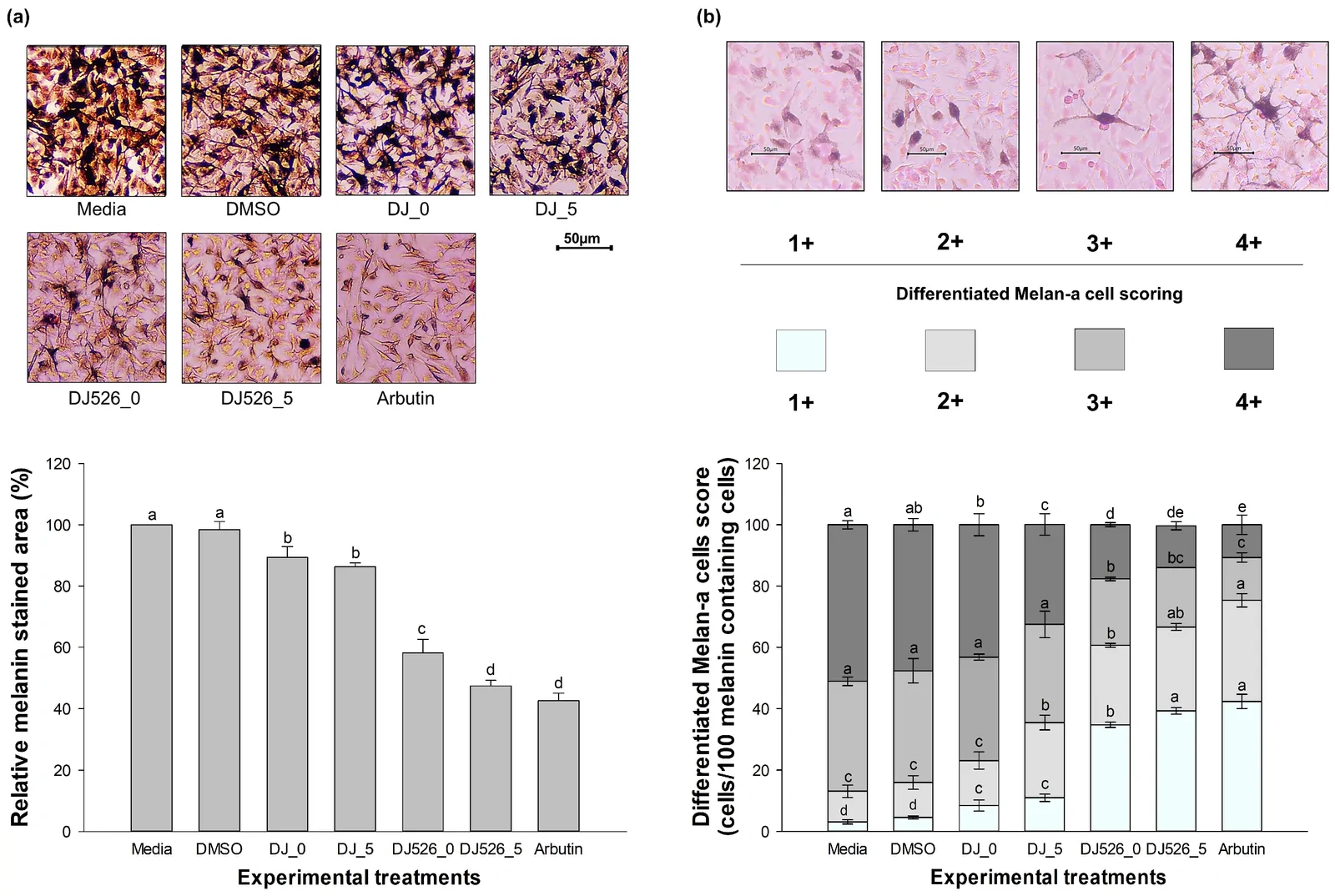
Effect of DJ526_5 treatments on in situ melanin content, morphology and pigmentation of Melan-a cells. (**a**) Relative melanin-stained area (%) of Fontana–Masson-stained Melan-a cells after treatment. (**b**) Morphological scoring and pigmentation assessment of Melan-a cells after treatment. The concentrations of DMSO and the treatments were 0.1% (*v*/*v*) and 100 µg/mL, respectively. Values are presented as mean ± SD. Different lowercase letters (a–e) indicate statistically significant differences among treatments (*p* < 0.05); “a” generally corresponds to the highest stained area or pigmentation, whereas letters b–e indicate progressively lower values.

To visually evaluate and quantify in situ melanin deposition, Fontana–Masson staining was performed alongside a 1+ to 4+ morphological grading assessment (Figure 3). Microscopic analysis revealed dense, heavily pigmented dark-brown argentaffin aggregates across nearly the entire cytoplasm in the vehicle and medium controls, corresponding to maximum relative melanin-stained areas (Figure 3a). This baseline pigment accumulation was significantly attenuated by the experimental treatments (*p* < 0.05). Notably, DJ526_5 emerged as the most potent inhibitor among the tested formulations, restricting the relative melanin-stained area to 47.37 ± 1.84%. This suppressive profile was statistically indistinguishable from the positive control, arbutin (42.55 ± 2.48%). DJ526_0 also displayed notable efficacy (58.17 ± 4.39%), significantly outperforming both DJ_5 (86.30 ± 1.31%) and DJ_0 (89.32 ± 3.58%).

This reduction in cellular pigment content was accompanied by shifted phenotypic differentiation profiles across a 100-cell population assessment (Figure 3b). While the control and DMSO groups were heavily dominated by mature, hyper-dendritic 3+ and 4+ phenotypes, treatment with DJ526_5 and arbutin increased the proportion of cells in the lower-pigmentation morphology category. Specifically, DJ526_5 increased the proportions of cells classified as grades 1+ (39.22 ± 1.07%) and 2+ (27.39 ± 1.13%), and reduced the proportion classified as grade 4+ phenotypes (13.67 ± 1.33%). This distribution closely mirrored the morphological profile of the arbutin group, sharing overlapping statistical significance markers across the morphological grades. Collectively, these findings demonstrate that DJ526_5 fundamentally amplifies anti-melanogenic capacity compared to the other tested groups, effectively disrupting melanocyte maturation and maintaining cells in a low-pigment, less mature state.

### 3.6. mRNA Expression of Melanogenesis-Related Genes

**Figure 4:**
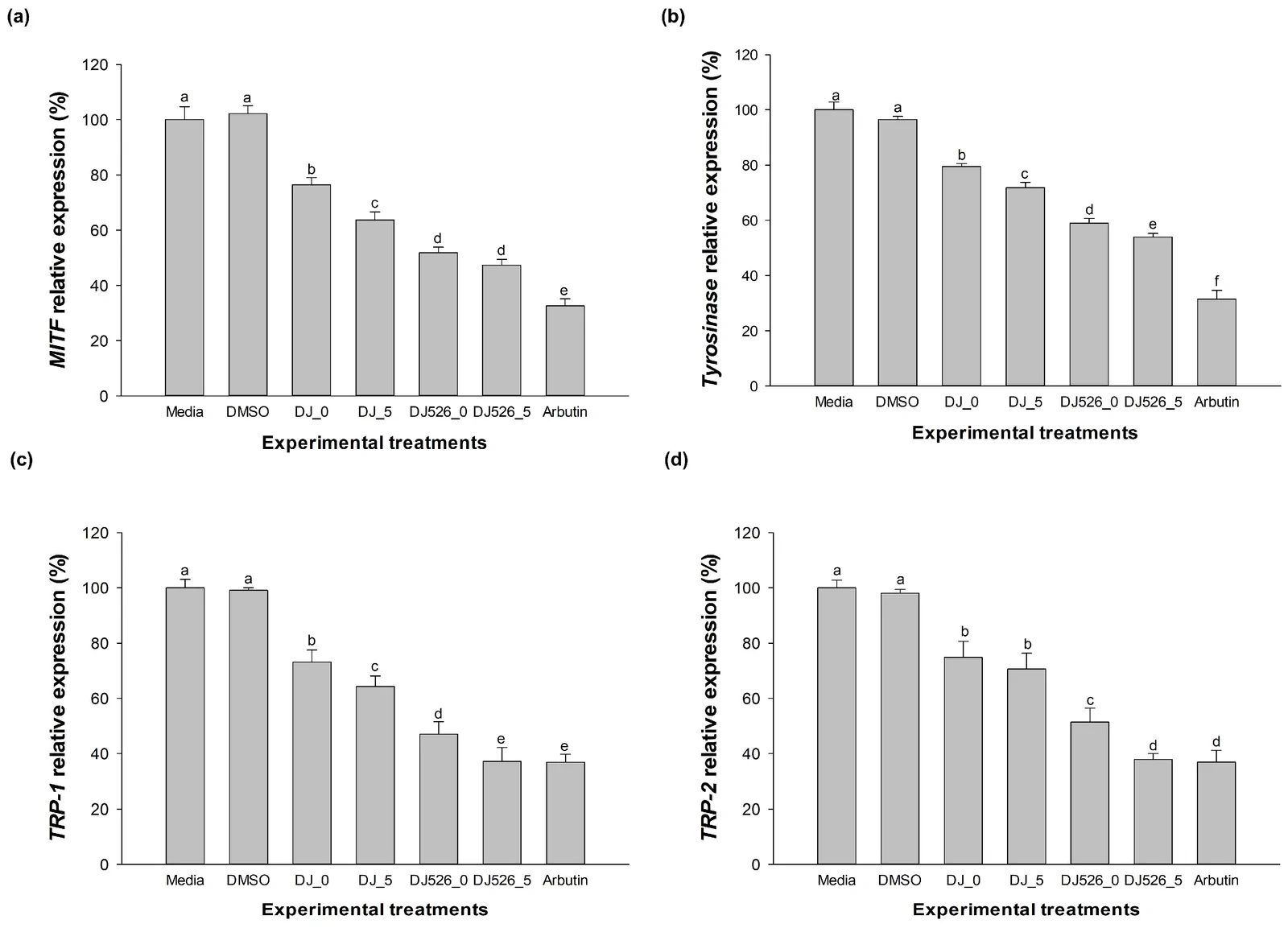
Effect of DJ526_5 treatment on relative expression of melanogenesis-related genes. (**a**) *MITF* expression levels; (**b**) *tyrosinase* expression levels; (**c**) *TRP-1* expression levels; (**d**) *TRP-2* expression levels. The concentrations of DMSO and the treatments were 0.1% (*v*/*v*) and 100 µg/mL, respectively. Values are presented as mean ± SD. Different lowercase letters (a–f) indicate statistically significant differences among treatments (*p* < 0.05); “a” generally corresponds to the highest value, whereas letters b–f indicate progressively lower values.

To elucidate the molecular mechanisms driving the enhanced anti-melanogenic activity of the resveratrol-enriched rice, we evaluated the transcriptional levels of the core regulatory genes—*MITF*, *tyrosinase*, *TRP-1*, and *TRP-2* (Figure 4). The transcription levels of these target genes are expressed relative to the untreated medium control, which was defined as 100%. While the vehicle control (0.1% DMSO) maintained baseline expression levels statistically indistinguishable from the medium control (*p* > 0.05), the tested treatments elicited varying degrees of transcriptional suppression. Remarkably, the 5-day germinated transgenic variant, DJ526_5, emerged as the most potent inhibitor among all experimental groups, demonstrating substantial suppressive effects across all targets and downregulating *MITF* and *tyrosinase* more effectively than the other formulations. Furthermore, the inhibitory effect of DJ526_5 on *TRP-1* and *TRP-2* expressions was particularly pronounced, driving transcript levels down sharply to closely match the performance of the positive control, arbutin. In comparison, the non-germinated transgenic rice (DJ526_0) and the parent controls (DJ_5 and DJ_0) exhibited a progressively weaker capacity to attenuate these transcripts. In summary, these findings substantiate that DJ526 and DJ possess inherent suppressive properties, but their germinated counterparts achieve a significantly more pronounced disruption of the melanogenic cascade in Melan-a cells.

### 3.7. Protein Expression Analysis of Melanogenesis-Related Targets

**Figure 5:**
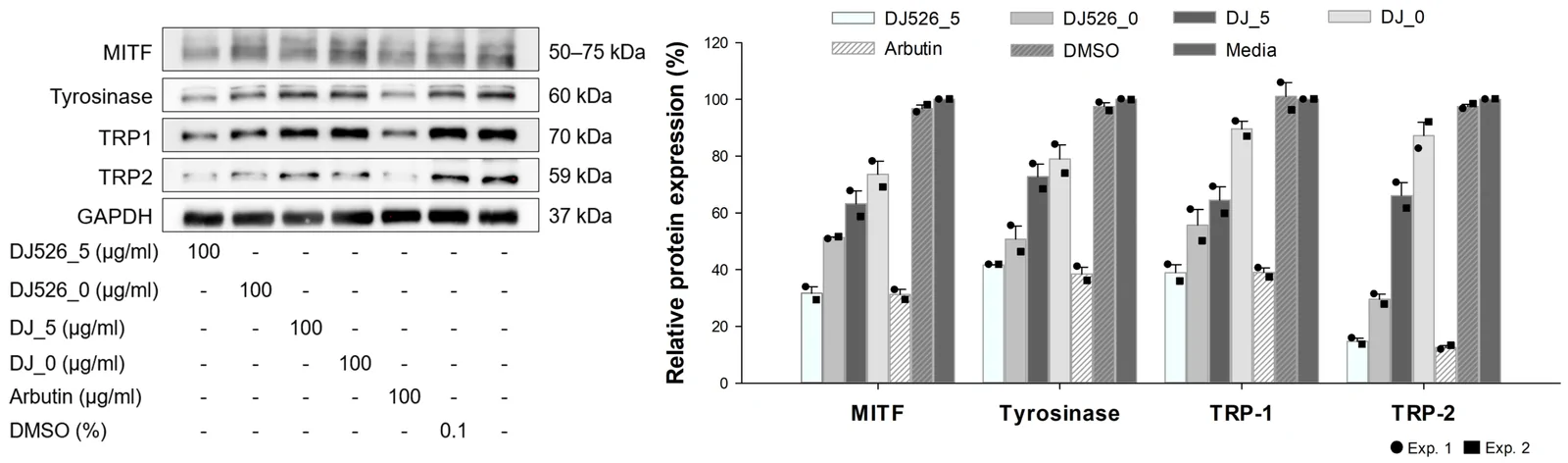
Effect of DJ526_5 treatment on protein expression levels of melanogenesis-related targets. The concentrations of DMSO and the treatments were 0.1% (*v*/*v*) and 100 µg/mL, respectively. “-” indicates that the corresponding treatment was not applied. Bars represent mean ± SD; symbols indicate individual experimental values from two independent experiments (*n* = 2).

The translational patterns of the master transcription factor MITF and its key enzymatic targets, tyrosinase, TRP-1, and TRP-2, were analyzed by Western blotting to trace the downstream molecular events regulating pigment synthesis (Figure 5). The vehicle (DMSO) and medium controls exhibited maximal protein expression levels, indicating a highly active baseline melanogenic cascade. However, exposure to the experimental treatments resulted in lower protein-expression levels of these melanogenic markers.

Among the evaluated groups, DJ526_5 exhibited the lowest expression levels of MITF protein (31.68 ± 2.33%), tyrosinase (41.66 ± 0.09%), TRP-1 (38.96 ± 2.82%), and TRP-2 (14.74 ± 1.08%). The protein-expression pattern of DJ526_5 was similar to that of arbutin across all four target proteins.

A clear downward trend in translational suppression was observed across the other treatment groups. DJ526_0 demonstrated intermediate regulatory control across the entire cascade. Conversely, both DJ_5 and DJ_0 exerted a progressively weaker impact on the cascade, allowing protein levels to remain elevated near baseline control levels. Overall, these protein-expression data from two independent experiments were consistent with the reduced melanin content and cellular tyrosinase activity observed in DJ526_5-treated Melan-a cells.

## 4. Discussion

Transgenic and postharvest strategies have been used to enhance the content and bioactivity of plant-derived phenolic compounds, including resveratrol-enriched rice DJ526 [47,51,61]. Within this context, the present study examined how a short germination period influences the functional properties of resveratrol-rich DJ526 rice in a melanocyte model.

The five-day germination period was selected based on published time-course evidence rather than preliminary optimization experiments. Previous studies in Iksan 526 (DJ526) reported that five days of germination increased trans-resveratrol and piceid contents by 1.7–2.1-fold and 1.4–3.5-fold, respectively, compared with ungerminated seeds. The piceid-to-resveratrol ratio was also approximately twofold higher on day 5 than on day 1, indicating a change in stilbene composition during germination [50,52]. Consistent with these reports, the present chemical profiling (Figure S2) showed that five-day germination increased piceid and trans-resveratrol contents in DJ526_5 relative to those in DJ526_0. The accumulation of trans-resveratrol and piceid during germination is likely associated with coordinated activation of resveratrol biosynthesis and glycosylation. Germination can enhance phenylpropanoid-pathway flux and increase the supply of *p*-coumaroyl-CoA, thereby supporting AhRS3-mediated resveratrol production. In the same DJ526 line, *AhRS3* transcript abundance increased from days 1 to 5 of germination and was highest at day 5, concomitant with increased trans-resveratrol and piceid contents [52]. Furthermore, candidate UDP-glycosyltransferases have been associated with piceid formation during DJ526 germination [50], while altered expression of *AhRS3*, *PAL*, *C4H*, *4CL*, and *UDP-glycosyltransferase* genes has been reported in developing Iksan 526 seeds compared with the non-transgenic Dongjin background [48]. Collectively, these findings support a mechanism in which germination promotes de novo resveratrol biosynthesis, followed by glycosylation to piceid. Although β-glucosidase-mediated piceid hydrolysis can contribute to the free-resveratrol pool, this process alone is unlikely to explain the simultaneous increase in both trans-resveratrol and piceid.

Oxidative stress and reactive oxygen species are recognized contributors to melanocyte regulation and pigment production, and antioxidant agents can modulate these processes by limiting redox-driven signaling inputs [62,63,64]. In this study, DJ526_5 showed the greatest ABTS radical-scavenging activity among the tested rice extracts, and its activity and VCEAC at 100 mg/mL were comparable to those of arbutin. The ABTS radical-scavenging assay was selected as a preliminary comparative method because it is widely used to assess the in vitro radical-scavenging capacity of plant-derived extracts and permits comparison among the rice extract samples. This finding indicates that germination strengthens the antioxidant capacity of the transgenic line. The enhancement observed in DJ526_5 is consistent with reports that seed germination reactivates primary and secondary metabolism, promotes the release and synthesis of phenolic compounds, and thereby modifies the antioxidant and bioactive profiles of cereal extracts [65,66]. Given the involvement of oxidative mechanisms in melanogenesis, this enhanced radical-scavenging activity may contribute to the downstream decreases in tyrosinase activity and melanin accumulation observed in Melan-a cells [5,67,68]. Previous studies on DJ526 primarily focused on germination-associated stilbene accumulation and its anti-inflammatory or anti-adipogenic activities in macrophage and adipocyte models [55,56]. Advancing beyond these reports, the present study establishes a novel application by demonstrating that this specific postharvest preparation strategy (5-day germination) directly translates to pronounced anti-melanogenic efficacy in Melan-a cells. Together with prior findings, the present data suggest that germination-associated changes in the stilbene pool and enhanced radical-scavenging capacity are distinct drivers of the newly observed suppression of melanin accumulation and tyrosinase activity, underscoring the novelty of DJ526_5 as a skin-lightening biomaterial. While the ABTS assay provided a useful preliminary comparison of radical-scavenging capacity among the extracts, complementary antioxidant assays based on different reaction mechanisms would further broaden the characterization of their antioxidant properties.

The functional consequences of germination were further evident in the Melan-a model. At the cellular level, DJ526_5 achieved a greater reduction in total intracellular melanin and functional tyrosinase activity than DJ526, without evidence of non-specific cytotoxicity. These quantitative data were supported by Fontana–Masson staining and morphological scoring, which showed a lower relative melanin-stained area increased the proportions of cells classified as grades 1+ and 2+ and decreased the proportion classified as grade 4+. Because dendrite formation and high pigment load are characteristic of active melanosome export, this shift suggests that DJ526_5 influences both melanin synthesis and melanocyte maturation, in line with studies reporting that resveratrol-based and rice-derived extracts can reduce dendricity and melanosome transfer in melanocytes [26,27,40,55]. These observations extend previous work showing that resveratrol-enriched rice and rice-derived extracts can combine antioxidant activity with suppression of melanogenesis in Melan-a cells and related models [46,51,55,61], and indicate that a short germination period further amplifies these anti-melanogenic effects.

The gene-expression results provide a molecular context for these cellular effects. MITF is a master regulator of melanocyte differentiation and melanin synthesis, coordinating the expression of tyrosinase and the tyrosinase-related proteins TRP-1 and TRP-2 [69,70,71]. In the present work, DJ526_5 markedly reduced the mRNA expression of *MITF*, *TYR*, *TRP-1*, and *TRP-2*, indicating suppression of the MITF-regulated melanogenic program. The protein-expression data from two independent experiments also showed a similar pattern, with lower levels of MITF, tyrosinase, TRP-1, and TRP-2 proteins. These findings are compatible with reports that resveratrol-enriched rice and resveratrol itself can downregulate MITF and downstream melanogenic enzymes and thereby reduce melanin synthesis [51,55,72,73]. Taken together with the reductions in cellular tyrosinase activity and melanin content, the results support suppression of the melanogenic pathway by DJ526_5. However, a cell-free tyrosinase assay was not performed, and direct inhibition of tyrosinase by DJ526_5 remains to be determined.

The non-transgenic parental controls help distinguish general germination-related changes in the rice matrix from changes associated with the resveratrol-enriched DJ526 background. Trans-resveratrol and piceid were not detected in DJ_0 or DJ_5, and although germination modestly improved the antioxidant capacity of DJ, both parental extracts showed weaker antioxidant and anti-melanogenic activity than DJ526_5. Previous compositional and targeted GC–MS comparisons also found DJ526 and Dongjin to be broadly comparable for measured grain components apart from the intended resveratrol-related difference [74,75]. Thus, the present results are consistent with a contribution of the germination-associated resveratrol/piceid increase to the enhanced activity of DJ526_5. However, the use of crude extracts precludes attribution of activity exclusively to these compounds. Germination may also change phenolic acids, flavonoids, and other rice-matrix constituents, which were not comprehensively measured in the present germinated samples. Further comparative phytochemical and metabolomic analyses are required to clarify the contributions of other constituents.

Resveratrol and piceid may make distinct contributions to the activity of DJ526_5. Piceid is the 3-O-β-D-glucoside of resveratrol, and glycosylation can influence solubility, chemical stability, cellular uptake, and biological activity. Resveratrol has established anti-melanogenic effects, including direct tyrosinase inhibition and suppression of MITF-associated signaling [27,30]. Although piceid can show weaker direct tyrosinase inhibition than resveratrol in some cell-free systems, it has also been reported to reduce cellular tyrosinase activity and melanin production [76,77,78]. Thus, the concurrent increase in resveratrol and piceid after germination may contribute to the enhanced activity of DJ526_5 through distinct or complementary mechanisms. However, because the present study used a crude extract, the observed antioxidant and anti-melanogenic effects cannot be assigned specifically to resveratrol, piceid, or their combined effects; other germination-responsive constituents may also contribute. In addition, piceid-to-resveratrol conversion was not examined in the Melan-a culture system. Although β-glucosidase-mediated piceid hydrolysis may occur in some biological settings, whether conversion occurred in the culture medium or within Melan-a cells under the present conditions remains unknown. Future studies comparing purified resveratrol and piceid, alone or in combination, and monitoring their levels after cell treatment may help clarify their individual contributions and potential bioconversion.

## 5. Conclusions

This study demonstrates that a five-day germination period significantly amplifies the bioactive profile of resveratrol-enriched DJ526 rice, accompanied by an increase in total resveratrol and piceid. Consequently, the germinated extract (DJ526_5) exhibits greater radical scavenging activity and profound, non-cytotoxic anti-melanogenic efficacy in Melan-a cells compared to its ungerminated and non-transgenic counterparts. By effectively reducing intracellular melanin content and cellular tyrosinase activity and suppressing the MITF-mediated melanogenic cascade, DJ526_5 comprehensively suppresses melanin synthesis. These findings establish germinated resveratrol-enriched rice as a promising functional biomaterial with significant potential for the development of cosmeceuticals targeting hyperpigmentation. However, because the present findings were obtained using crude extracts in an in vitro Melan-a cell model, future work should define the respective roles of trans-resveratrol and piceid and assess efficacy and safety in in vivo models.

## Figures and Tables

**Table 1:** Differentiated Melan-a cell scoring system.

Score	Morphology and Pigmentation Criteria		
	Cell Shape/Dendrites	Cell Size (µm)	Pigmentation Pattern
1+	Dendritic, bipolar spindle	1–50	visible Fontana–Masson-positive pigment occupying <50% of cytoplasmic area
2+	Dendritic, bipolar spindle	1–50	visible Fontana–Masson-positive pigment occupying ≥50% of cytoplasmic area
3+	Multipolar spindle without tip branches	>51	Pigmentation throughout the cytoplasmic area
4+	Multipolar spindle with numerous spindle branches	>51	Pigmentation densely packed throughout the cytoplasmic area

**Table 2:** ABTS radical scavenging activity, vitamin C equivalent antioxidant capacity and IC_50_ of each treatment.

Treatments	Stock Concentration (mg/mL)	ABTS Radical Scavenging Activity (%)	Vitamin C Equivalent Antioxidant Capacity (mg VCE/mL)	IC_50_ (mg/mL)
DMSO	5% (*v*/*v*)	3.97 ± 0.24 ^e^	ND	
DJ_0	10	8.50 ± 0.14 ^c^	0.008 ± 0.000 ^c^	>100 mg/mL
	25	12.37 ± 0.20 ^d^	0.018 ± 0.001 ^d^	
	50	18.13 ± 0.36 ^d^	0.034 ± 0.001 ^d^	
	100	33.35 ± 0.48 ^d^	0.077 ± 0.001 ^d^	
DJ_5	10	7.66 ± 0.04 ^c^	0.005 ± 0.000 ^c^	>100 mg/mL
	25	16.24 ± 0.30 ^c^	0.029 ± 0.001 ^c^	
	50	26.33 ± 0.56 ^c^	0.057 ± 0.002 ^c^	
	100	39.66 ± 1.77 ^c^	0.094 ± 0.005 ^c^	
DJ526_0	10	31.12 ± 0.68 ^b^	0.070 ± 0.002 ^b^	21.34 ± 0.56
	25	54.80 ± 0.79 ^b^	0.136 ± 0.002 ^b^	
	50	69.62 ± 0.15 ^b^	0.177 ± 0.000 ^b^	
	100	84.02 ± 2.33 ^b^	0.218 ± 0.006 ^b^	
DJ526_5	10	35.28 ± 1.21 ^a^	0.082 ± 0.003 ^a^	16.81 ± 1.26
	25	59.97 ± 2.81 ^a^	0.151 ± 0.008 ^a^	
	50	80.99 ± 3.36 ^a^	0.209 ± 0.009 ^a^	
	100	93.17 ± 0.18 ^a^	0.243 ± 0.000 ^a^	
Arbutin	100	93.17 ± 0.06 ^a^	0.243 ± 0.000 ^a^	

Results are expressed as mean ± standard deviation (*n* = 3). Different lowercase letters (a–e) within the same concentration indicate statistically significant differences among treatments (*p* < 0.05); “a” generally corresponds to the highest antioxidant activity at that concentration, whereas letters b–e indicate progressively lower activities. “ND” indicates that the calculated VCEAC value was below zero after conversion using the vitamin C standard curve. An IC_50_ value of “>100 mg/mL” indicates that 50% inhibition was not reached within the tested concentration range.

## Data Availability

The original contributions presented in this study are included in the article/Supplementary Material. Further inquiries can be directed to the corresponding author.

## Supplementary Materials

The following supporting information can be downloaded at: https://www.mdpi.com/article/10.3390/cimb48101004/s1.

## Abbreviations

The following abbreviations are used in this manuscript:

ABTS	2,2′-azinobis (3-ethylbenzothiazoline-6-sulfonic acid)
DMSO	Dimethyl sulfoxide
L-DOPA	L-dihydroxy phenylalanine
MITF	Microphthalmia-associated transcription factor
RPMI	Roswell Park Memorial Institute
TRP-1	Tyrosinase-related protein 1
TRP-2	Tyrosinase-related protein 2
VCEAC	Vitamin C equivalent antioxidant capacity
