# Supplementary material for: Germination Improves the Antioxidant and Anti-Melanogenic Properties of Resveratrol-Enriched DJ526 Rice in Melan-a Cells
Source: Curr Issues Mol Biol. 2026 Sep 29;48(10):1004. doi: 10.3390/cimb48101004 (PMC13703750; doi:10.3390/cimb48101004)
Supplement: Supplementary file 1 [file cimb-48-01004-s001.zip › cimb-4558224-supplementary.pdf]

Supplementary materials

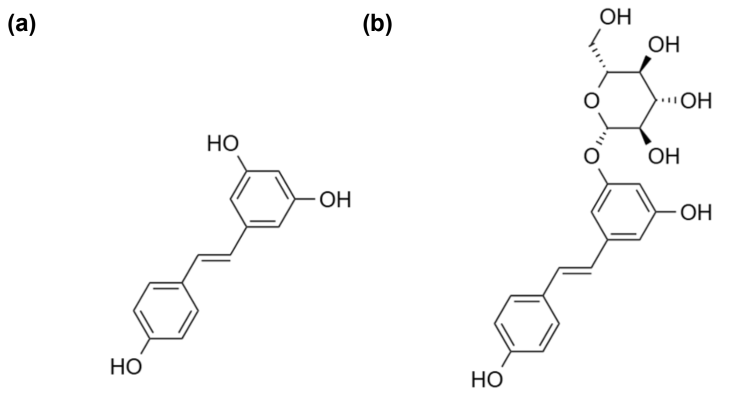

Figure S1. Chemical structures of the major resveratrol-related stilbenes in resveratrol-enriched rice: **(a)** *trans*-resveratrol and **(b)** *trans*-piceid (polydatin; trans-resveratrol-3-O-β-D-glucoside).

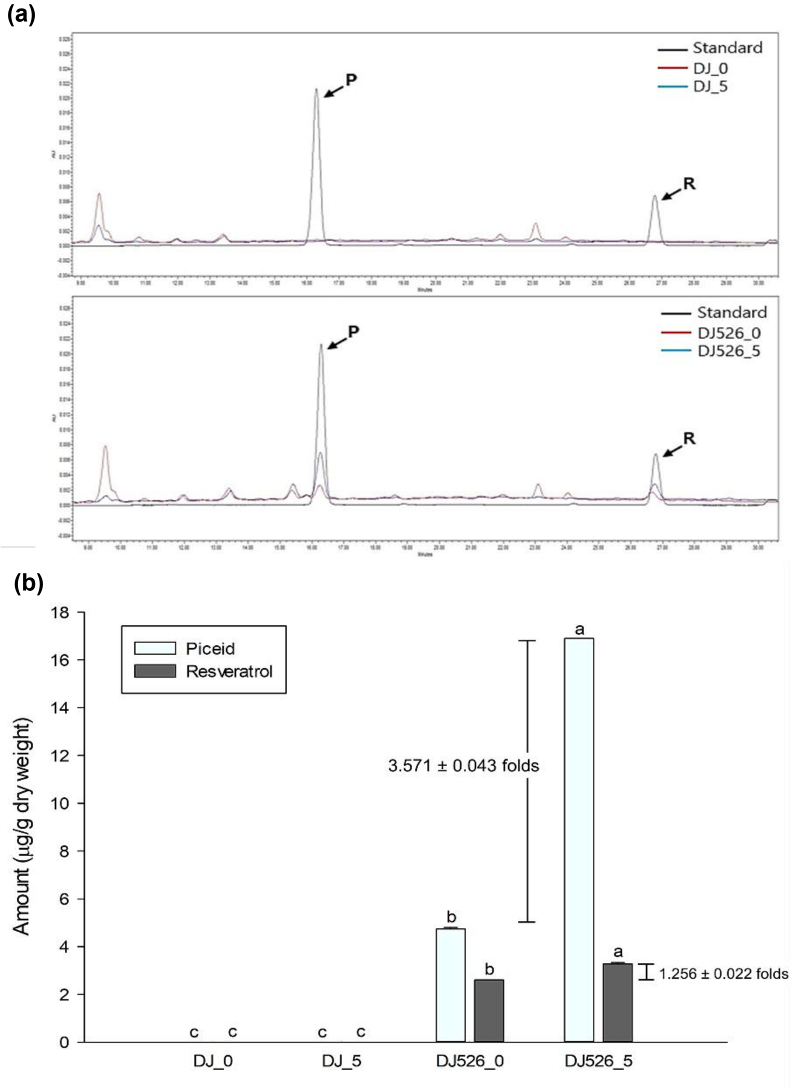

Figure S2. Piceid and resveratrol amounts in extracts. **(a)** Representative High-performance liquid chromatography (HPLC) electropherograms of the standard mixture and extracts. Peaks corresponding to piceid and resveratrol are indicated by **P** and **R**, respectively. **(b)** Piceid and resveratrol concentrations, expressed as µg/g dry weight, were quantified using the respective standard calibration curves. Values are presented as mean ± SD. Different lowercase letters (a–c) indicate statistically significant differences among treatments ($p < 0.05$); "a" generally corresponds to the highest piceid or resveratrol amount, whereas letters b–c indicate progressively lower amounts.

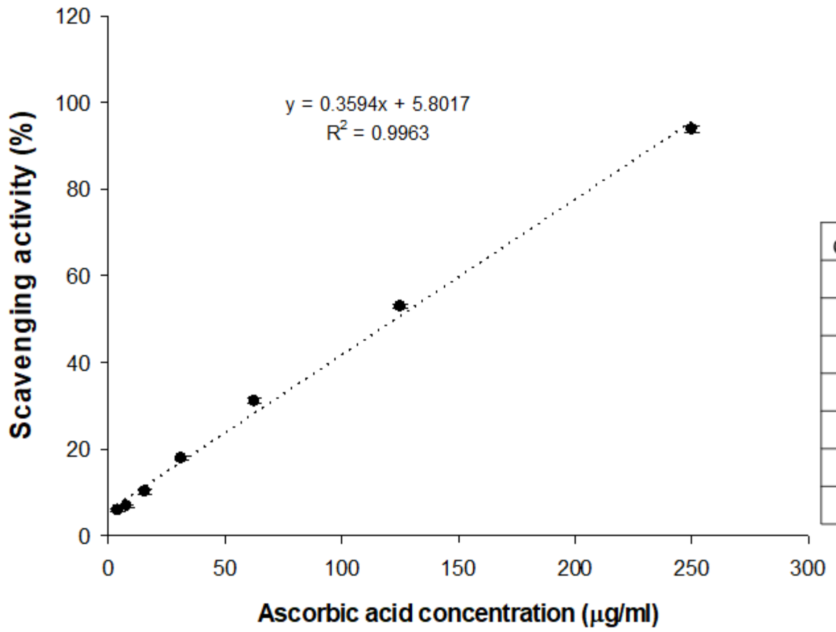

Concentration	Mean Inhibition (%)
250.00	93.85
125.00	52.99
62.50	31.10
31.25	17.91
15.63	10.24
7.81	6.90
3.91	5.92

Figure S3. Ascorbic acid (vitamin C) standard for VCEAC determination.

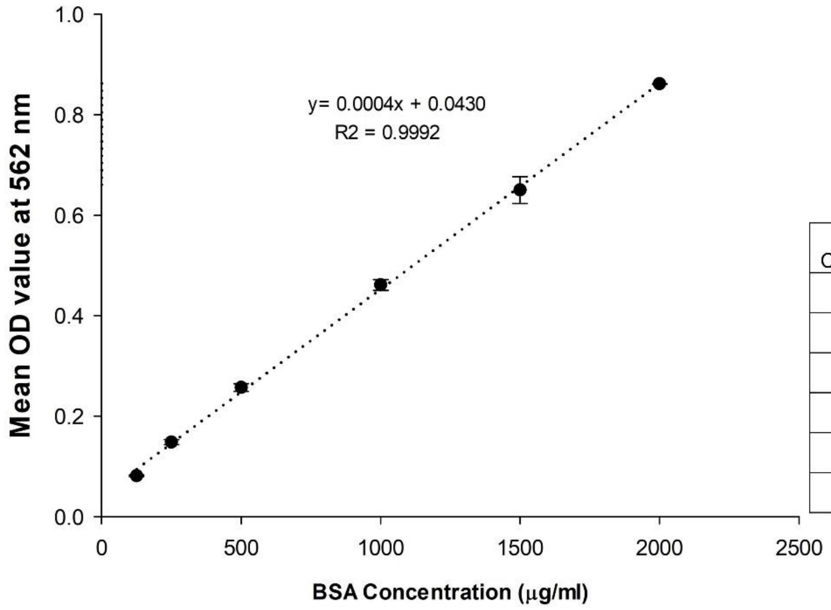

Concentration	Mean OD value
2000	0.862
1500	0.650
1000	0.462
500	0.257
250	0.148
125	0.082

Figure S4. BSA calibration curve for protein quantification.

Table S1. The sequence of each primer used for RT-qPCR.

Gene	Accession number	Sequence (5'−3')	Target size (bp)
MITF	NM_001113198.2	FP: AGC GTG TAT TTT CCC CAC AG<br>RP: CCT TAG CTC GTT GCT GTT CC	239
Tyrosinase	D00131.1	FP: CCA GAA GCC AAT GCA CCT AT<br>RP: CCA GAT ACG ACT GGC CTT GT	193
TRP-1	NM_031202.3	FP: TCT GGC CTC CAG TTA CCA AC<br>RP: TCA GTG AGG AGA GGC TGG TT	223
TRP-2	X63349.1	FP: ACC CTG TGT TTG TGG TCC TC<br>RP: GTT GCT CTG CGG TTA GGA AG	186
GAPDH	NM_001289726.2	FP: AAC TTT GGC ATT GTG GAA GG<br>RP: ACA CAT TGG GGG TAG GAA CA	223

Note: FP represents the forward primer and RP represents the reverse primer.
